# Guidelines for the identification and distribution of patient samples in the medical laboratory

**DOI:** 10.1155/S1463924694000027

**Published:** 1994

**Authors:** P. A. Bonini, N. Alpert, M. Luzzana, R. Rubin

**Affiliations:** 1 lstituto Scientco H. San Raffaele Laboratorio Analisi Via Olgettina 60 Milano 20132 Italy; 2 Instrument Consultation P.O. Box 3403 447 Colenbrook Road Stamford CT 06906 USA; 3 Consiglio Nazionale delle Ricerche Via Ampere 56 Milano 20100 Italy; 4 43218 Pauline Drive Chevy Chase MD 20815 USA


Journal of Automatic Chemistry, Vol. 16, No. 1 (January–February 1994), pp. 25–32

### Guidelines for the identification and distribution of patient samples in the medical laboratory

### Recommendation of the European Committee for Clinical Laboratory Standards (ECCLS) and approved document of the International Federation of Clinical Chemistry (IFCC)

**P. A. Bonini[1], N. Alpert[2], M. Luzzana[3] and R. Rubin[4]**
[1]*Istituto Scientifico H. San Raffaele, Laboratorio Analisi, Via Olgettina 60, 20132 Milano, Italy;*
[2]*Instrument Consultation, P.O. Box 3403, 447 Colenbrook Road, Stamford, CT 06906, USA;*
[3]*Consiglio Nazionale delle Ricerche, Via Ampere 56, 20100 Milano, Italy;*
[4]*3218 Pauline Drive, Chevy Chase, MD 20815, USA*

### 1. Introduction

Diagnostic activities are performed:

(1) *In vivo*, with a patient as direct object of the investigation.

(2) *In vitro*, by the examination of a patient sample.

A major problem for *in vitro* procedures is to ensure the attribution of the results of all examinations to the correct patient.

This paper provides standards of terminology in accord with internationally acceptable definitions; criteria for optimal systems pertinent to the multiplicity of health care delivery systems; a description of current and developing modalities for patient/service linkage; requisite criteria for optimal systems and guidelines for their assessment.

Using current technologies as examples does not preclude the introduction of new technologies, for example magentic, optical or DNA fingerprinting devices, to perform the same functions in the future.

### 2. Terminology

2.1. *List of terms*

2.1.1. *Sample*: Material available for investigation. This term often needs to be qualificated in order to avoid ambiguity.

2.1.2. *Patient sample*: Patient material available for investigation.

2.1.3. *Fractionated sample*: The representative part of a patient sample which is separated for investigation.

2.1.4. *Sample aliquot*: The amount or volume of the fractionated sample taken for a procedure.

2.1.5. *Identification*: Data on, or in sample containers pertinent to patient identification and test order.

2.1.6. *Patient data*: Information which allows patient identification (basic data) and is helpful to the laboratory staff in the diagnostic process and in laboratory management (additional data).

2.1.7. *Request*: The list of tests requested for the patient. Normally, this is accompanied by patient data.

2.1.8. *Direct (positive) identification*: The identity of the patient is unequivocally linked to the sample container at the collection of the sample, and the linkage is maintained throughout the analytical process, including the reporting step.

2.1.9. *Indirect identification*: The patient sample, the test sample and/or the test portion is identified by its location (2). (Modified definition.)

2.2. *Comments*

2.2.1. *Basic patient data*: Consist of a permanent ID number, full name, sex and birth date (or age).

2.2.2. *Additional patient data*: May include the patient's address or that or relatives; patient's location in the medical facility; physician's name; relevant clinical data concerning the patient; and information about the patient's insurance.

2.2.3. *Test coding*: Each test is defined by the name of the component or the physical property to be determined: in the case of potential ambiguity, the sample type (for example serum, whole blood, urine, or tissue) should be specified.

2.2.4. *Labelling*: For infectious patient samples an appropriate warning should be present on the sample container.

2.2.5. *Request* The following information should also be included.

(*a*) Address to which results should be delivered

(*b*) Clinical information relevant to the request (for example presumptive diagnosis, diet, month of pregnancy)

(*c*) The time or timing of the sample collection; its volume; additives; special collection conditions; collection site; transport and storage

© 1994 IFCC. SD-CAS-ECCLS/1

procedures, when relevant for the interpretation of test results.

2.2.6. *Direct (positive) identification*: The terms 'positive identification' and 'positive sample identification' are often used to describe the automatic association, in an analytical instrument, of the tests results to an identified sample or sample aliquote. Direct identification has a more extended meaning in this document. The unequivocal linkage of patient, sample and reporting results must be included in the process.

### 3. Patient, physician, laboratory and administration

The following generalized diagrams illustrate various functional modes of the laboratory in systems of patient care.

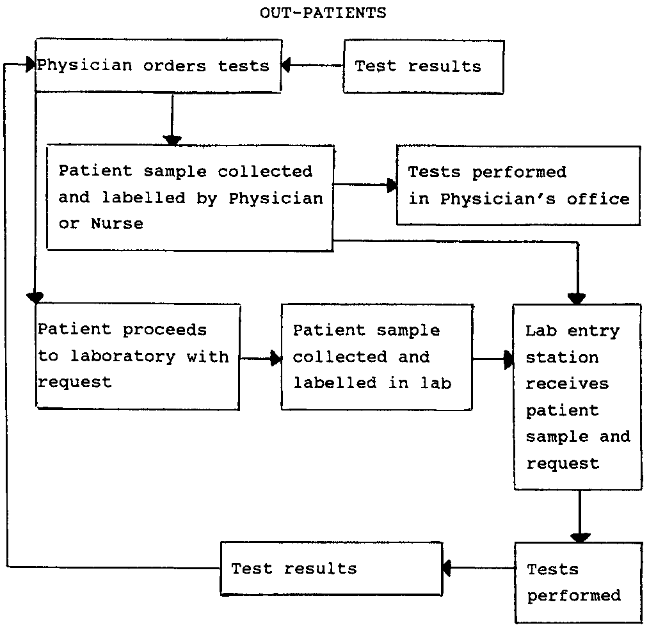

*Diagram 1*

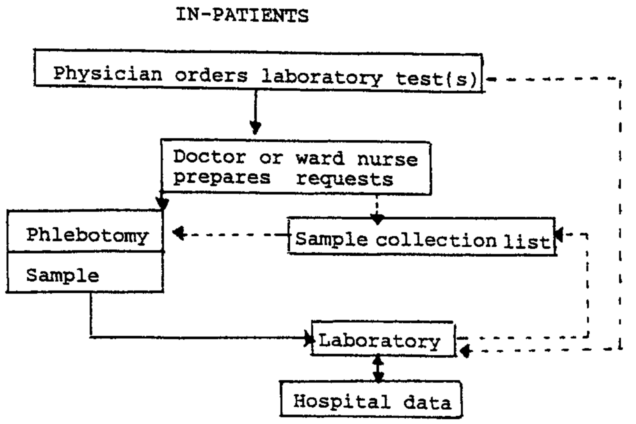

*Diagram 2*

### 4. Sample identification procedures

### 4.1. *Manual system*

4.1.1. *MS1 basic manual system (see figures 1 and 2)*

The patient sample is obtained without identification or with only minimal identification. The request, written manually or imprinted from a plastic card embossed with identification at the health care facility, may be wrapped around the sample. Upon arrival at the laboratory, the patient sample and request are given matching numbers. The results of analysis of a single determination or group of determinations, which can be performed without splitting into sample aliquots or worksheet requirement, are noted on the request.

4.1.2. *MS2 manual system requiring splitting into test samples and worksheets*

Patient samples with identification, sample aliquots and requests written manually (figures 3 and 4) or by imprinting with identification from an embossed card (figures 5 and 6), are given matched numbers. Sample aliquots and worksheets listing required tests and patient sample numbers are distributed to work stations in the laboratory. Analytical results are entered on the worksheets which are gathered, collated and transcribed on the request or on patient report forms.

### 4.2. *Automated system*

4.2.1. *AS1 basic automated system*

Entry of the physician's request at the computer visual display terminal (VDT) prompts the printing of adhesive-backed labels with identification and essential request information to be attached to the sample container. Printing of a request can be an option (see figures 7 and 8).

Alternatively, a package of preprinted, adhesive-backed labels with patient identification, is provided at the hospital admission (see figures 9 and 10). When tests are requested, additional information (for example test[s]

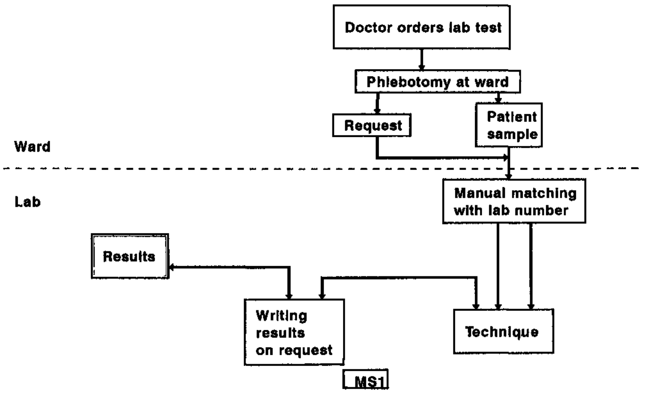

*Figure 1. MS1—schematic representation. HIS = Hospital Information System; LIS = Laboratory Information System.*

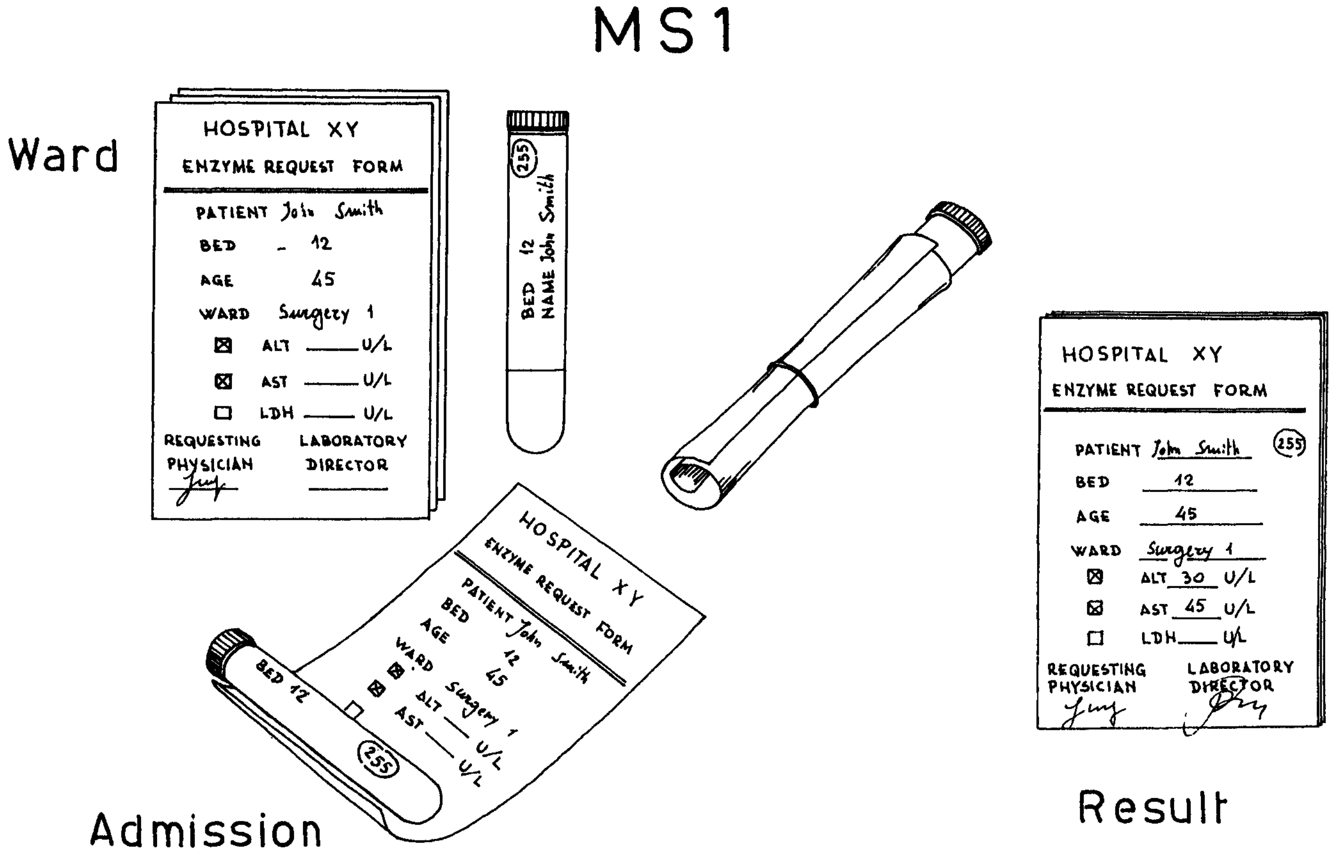

*Figure 2. MS1—pictorial representation.*

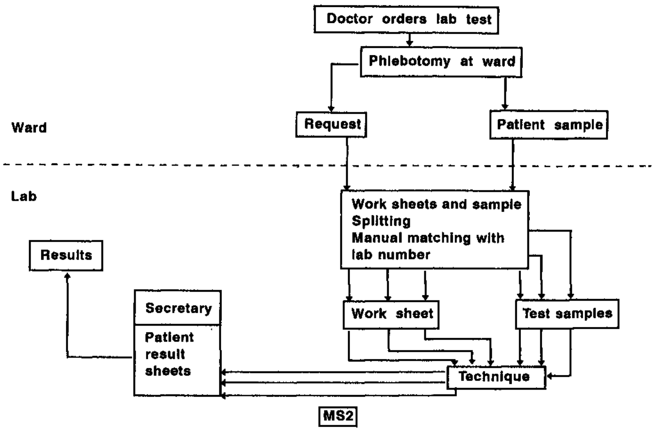

*Figure 3. MS2—schematic representation.*

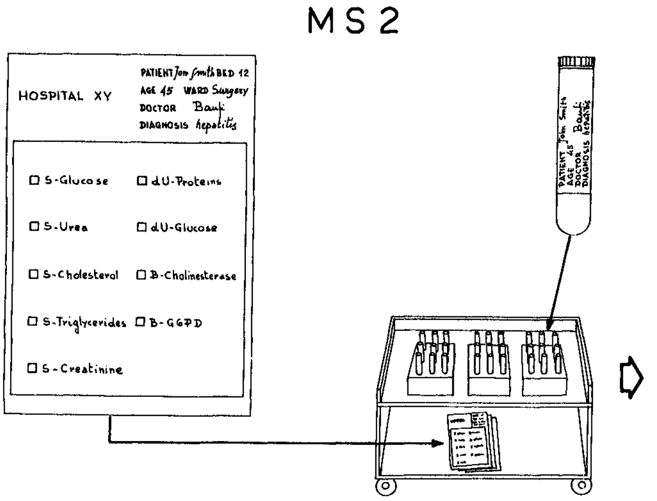

*Figure 4. MS2—pictorial representation.*

requested, date) is, if necessary, added and usually written on the labels.

After collection of the sample from the patient and its delivery to the laboratory, the entry of the identication and/or request generate accession-numbered adhesive labels for required sample aliquots and correlation with the patient sample, worklists and request, if required.

Completion of the procedure at the work stations can provide the results as an instrument printed output in a sequence correlated with a sample position in the process, or may be entered manually on the worksheets.

Collation of the worksheet data and its entry into the laboratory VDT generates the patient result report for delivery to the physician.

Some automated instruments are capable of performing all the requested tests on the original patient sample without being split into test samples. This simplifies the overall processing since an instrument printed analytical result can be correlated with the sample's sequential position in the system. The data may then be processed as above for reporting to the physician.

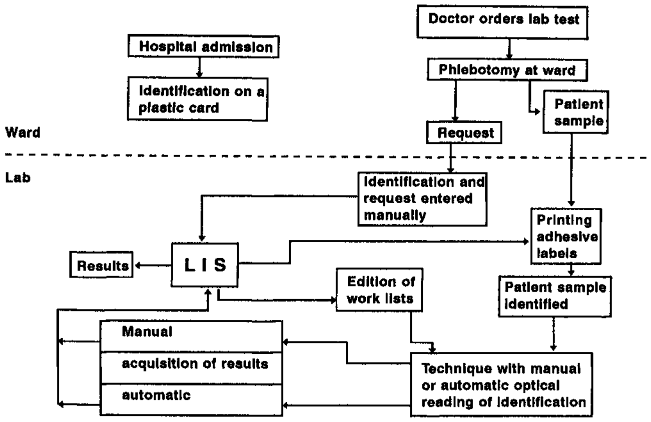

*Figure 5. MS2 with plastic card—schematic representation.*

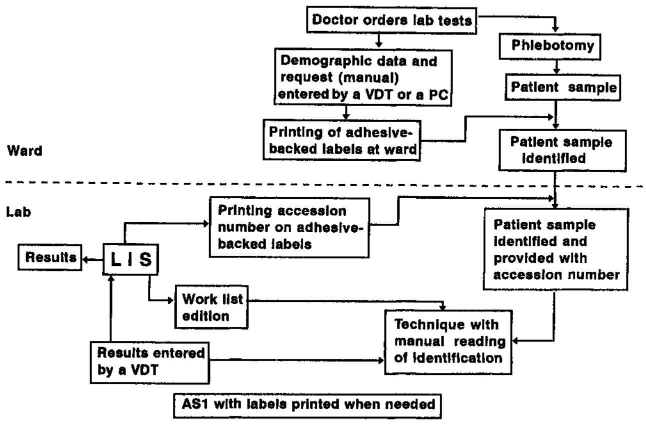

*Figure 7. AS1 with labels printed when needed—schematic representation.*

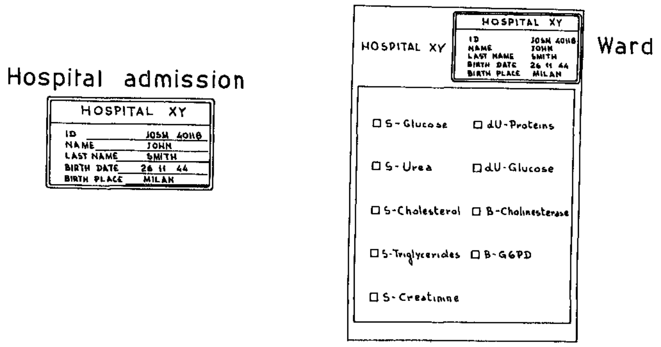

*Figure 6. MS2 with plastic card—pictorial representation.*

4.2.2. *AS2 Automated system with machine-readable identification* (*see figures 11 and 12*)

Present technology allows the increasing utilization of the combination of machine- and man-readable identification. Thus the scanning of the machine readable bar-coded identification component of an embossed patient card, combined with scanning of a bar code identified test request menu, provides a facile automated data entry method for the computer generation of patient sample container labels, requests, test sample labels and worksheets if required for processing.

Bar-coded identification is printed on patient sample and

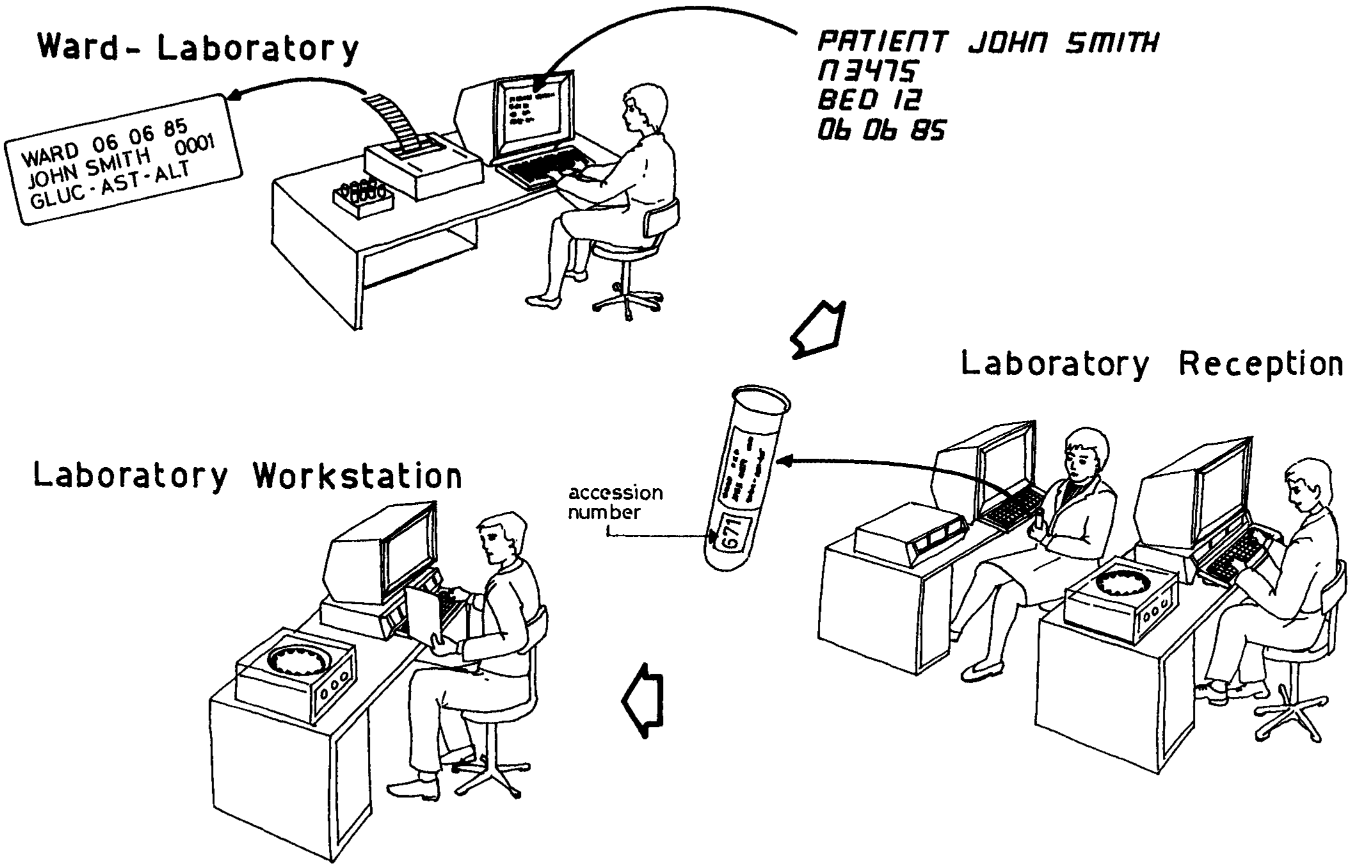

*Figure 8. AS1 with labels printed when needed—pictorial representation.*

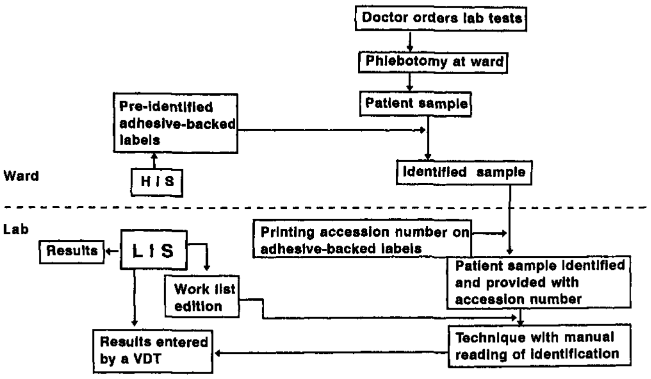

*Figure 9. AS1 with preprinted labels—schematic representation.*

test sample labels, and, if necessary, on requests and worksheets. Bar-code scanners at work stations permit the transmission of patient sample identification combined with the analytical results to the computer for generation of reports to the physician. The utilization of handheld bar-code scanners allows adaptation to manual work stations and semi-automated analytical units.

4.2.3. *AS3 automated systems with direct (positive) patient identification (see figures 13 and 14)*

There are two systems currently in use which allow direct linkage between patient and sample or service attainable at the time of sample collection or provision of service. The patient is provided with a wrist bracelet or other attachment with man-readable and bar-coded identification. When using a pre-labelled patient sample container as described in section AS2, a reading with a hand-held scanner of the bar-code identification attached to the patient and of the bar-code identification on the patient sample container, provides a visual display of the concordance, or the absence, of the identity. In addition, a permanent record of the transaction is obtained simultaneously, which can be down-loaded into a computer on demand.

An alternative process provides a man- and machine-readable copy of the patient-attached identification by transfer of this information onto an adhesive-backed label, which can be placed on the sample container while the patient sample is being collected. A transaction record can be achieved as described above.

With either method the process of the provision of patient services, whether analytical or of another kind, can be completed without manual recording or data transcription from the entry of patient into the health facility to the delivery of the service. In addition there is a verifiable audit trail of each step in the processing.

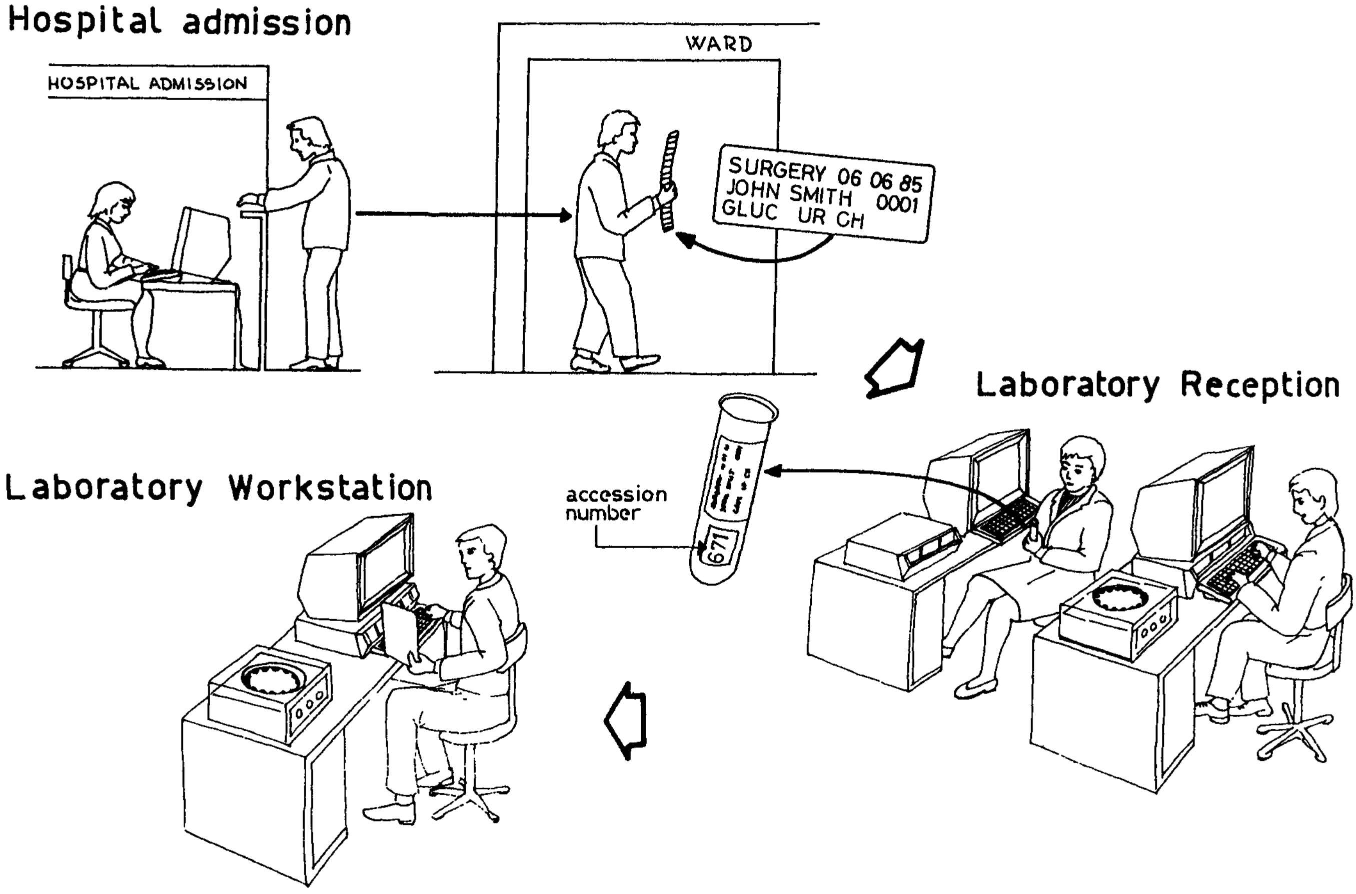

*Figure 10. AS1 with preprinted labels—pictorial representation.*

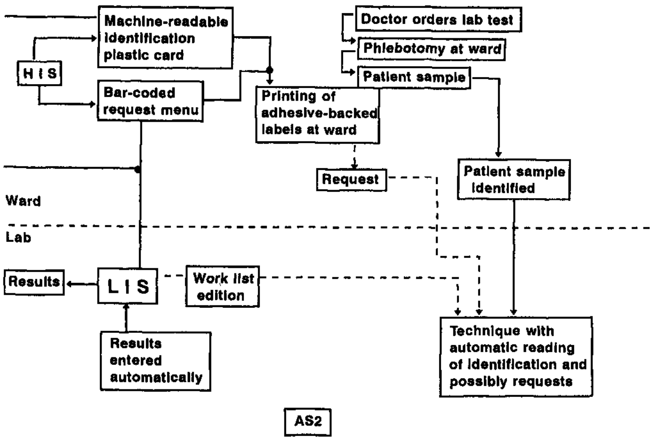

*Figure 11. AS2—schematic representation.*

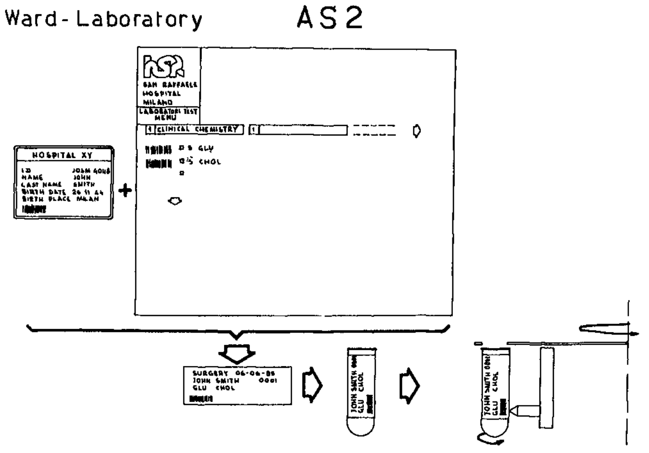

*Figure 12. AS2—pictorial representation.*

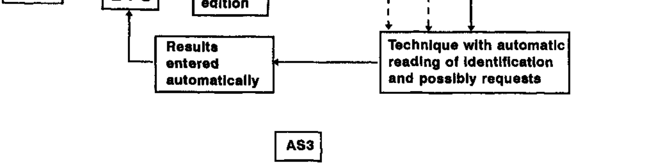

*Figure 13. AS3—schematic representation.*

### 5. System implementation and assessment

### 5.1. *Implementation*

Each step of the system in current use should be examined and documented in all aspects, from the point of entry of the patient to the medical facility, through to the request

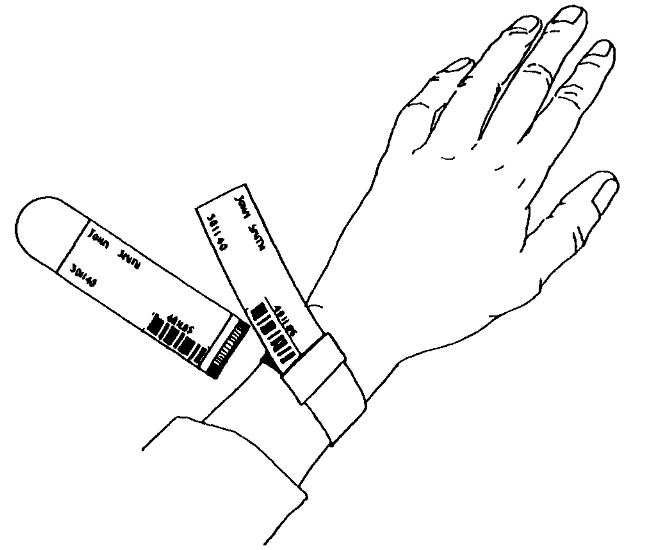

*Figure 14. AS3—pictorial representation.*

process, the analytical sequence and reporting of results. All ancillary aspects of personnel involvement and record maintenance are noted. The turn-around time from test request to test report is also established. The result is a classic time and motion study, which can be used as a base-line for a status review of present operations and comparison with proposed modification.

With an available documentation and analysis of the system in use it is possible to delineate the objectives, modalities, costs and possible benefits of change. In such a process the specific institutional and environment needs must be given adequate consideration. Following the analysis, planning and decision stages, the implementation of system modifications requires the development of a timetable for installation with detailed scheduling for the material requirements and intensive staff instruction.

The subsequent conduct of the effort requires monitoring for its implementation and function followed by a repeatable time and motion study, as well as an evaluation of the consequences of changes on patient care.

Major emphasis must be placed upon the availability of back-up procedures and instructing staff in their use to provide safe patient care in the event of difficulty in the implementation of procedural changes.

### 5.2. *Assessment criteria*

A list of 'yes'/'no' questions follows which is essential for assessing an identification system. An 'X' under 'yes' or 'no' is the answer for an optimal system. If there is no 'X' then it is uncertain whether 'yes' or 'no' is better. An 'X' under 'yes' or 'no' is counted as a definite answer; when uncertain, i.e. advantages and disadvantages are connected either to 'yes' or to 'no', no X is given (see section 5.3).

| | | Yes | No |
|---|---|---|---|
| (1) | Is the matching of patient sample identification and the patient automated? | X | |
| (2) | Does the system allow the patient to check the identification on the patient sample container? | X | |
| (3) | Does the patient sample carry human- and machine-readable identification? | X | |
| (4) | Does the patient sample container report the identification of the patient and of the patient sample? | X | |
| (5) | Does the patient sample container report the tests requested? | | |
| (6) | Does the system allow samples to be fractioned for delivery to specialized laboratories or laboratory sections? | X | |
| (7) | Are requests automatically delivered to specialized laboratories or sections of the laboratory? | | |
| (8) | Does the system require centralized patient sample reception and handling? | | |
| (9) | Does the system require centralized collation of patient data, requests and results? | | |
| (10) | Are spare parts available for all system components? | X | |
| (11) | Is system backup available for instrumentation programming communication linkages and computers? | X | |
| (12) | Is a direct linkage provided between: | | |
| | (*a*) Identification of the patient and generation of labels for patient samples? | X | |
| | (*b*) Generation of labels for patient samples and their location on the patient sample container? | X | |
| | (*c*) Affixing labels on the patient sample containers and collecting the blood? | X | |
| | (*d*) Generation of labels for fractionated sample containers, affixing them on the tubes and transferring fractionated samples? | X | |
| (13) | Is system backup available for automatically matching identification on pre-labelled containers and patient data reported on a wrist-bracelet or other attachment, and downloading the record of the transaction? | X | |
| (14) | Is the system expandable and compatible with other (for example pharmacy, radiology, blood banking etc.) components of the healthcare delivery system? | X | |

### 5.3. *Comments*

Criterion (2) refers to automated systems without direct (positive) patient identification; a facility for the patient to check the identification on the patient sample containers (the name, at least, must be clearly printed) can reduce identification errors. This is especially useful for out-patients.

Criterion (4) is particularly important for the tests requiring collection of blood at different times, from the same patient (for example glucose tolerance test).

Criterion (5): advantages of 'yes'—the reliability of providing backup for the information already present on the request is improved. In addition, patient samples can be processed as soon as they arrive at the laboratory. Disadvantages: the size of the label could be too large for standard tubes.

The support mentioned in the criterion (6) is essentially represented by the automatic generation of labels for fractioned samples containers, when neeeded.

Criteria (7), (8) and (9): advantages of answering 'yes' to criterion 7 and 'No' to the criteria 8 and 9—it is usually accepted that errors increase with the number of operations required. Thus, if a section of the laboratory is loaded only with its share of the work and no central reception is required, the consequent reduction in the number of operations should result in a reduced number of errors. Disadvantages: the existence of a central laboratory reception can actually speed the handling of patient samples through ease and rapidity of checking the patient sample, its identification, entry of its receipt into the information system, separation of serum, if necessary, and batching samples for distribution to work stations. This is especially important for the systems MS2 and AS1.

Criterion (10) applies to automated systems which should be planned for fault tolerance.

Criterion (11) states that the optimal system should provide integration of patient sample identification and input programming of analysers.

Criterion (12) states that in an optimal system:

(*a*) Production of labels should be activated by, and immediately follow, patient identification.

(*b*) Labelling of patient sample containers should automatically follow the production of labels.

(*c*) Blood collection should only be possible immediately after labelling the patient sample.

(*d*) Fractioning samples should only be possible immediately after generation of labels for fractionated samples and their location on fractionated samples tubes.

If the answers for criterion (12) (*a*), (*b*) and (*c*) are 'Yes', then criterion (13) is of no importance; if the answer is 'No', then criterion (13) offers an alternative for an optimal system. This alternative is now feasible.

### 6. Criteria for optimal systems

### 6.1. *Security*

For a patient sample identification system to be be really safe (free of errors), it must not depend on the good will of operators, but on necessary requirements in all relevant operations. This means that if any step of the procedure is not correctly followed, the system should stop operations automatically. If such a procedure is not technically feasible, an acceptable level of safety must be an integral component of the system in which it is always possible to check if all the operations have been correctly performed. This means that, at any point in the linkage of the patient, sample, sample processing and result report, or other service where identification is at risk, the system should ensure its internal security. The following recommendations concern security:

#### 6.1.1. *At collection phase:*

(1) Labels should be automatically generated as a result of a request, printed by a device located near the patient, just before venepuncture, obligatorily activated by an identification worn by the patient (for instance a wrist-bracelet with patient data) or strictly linked to his body. Alternatively, if labelled blood collection tubes are prepared in advance, patient identification reported on patient sample containers should be automatically matched, at the time of venepuncture, with patient data reported on a wrist-bracelet or other device attached to the patient's body. This transaction must be automatically recorded.

(2) Labels should include, in addition to basic patient data, the date (month and day) and time of their printing. This allows the laboratory to check, within reasonable limits, the temporal correspondence between label production and venepuncture. In addition, if labels are printed just before venepuncture, as recommended, the indication of date and time of their printing could eliminate the need of renumbering the patient sample containers (and possibly the requests) at their accession to the laboratory, with an 'accession number', usually adopted, in addition to basic patient data, to identify a single collection event. The permanent ID number, at least, should be machine-readable.

(3) Sufficient tubes should be used to avoid, when possible, subsequent splitting the patient sample into fractionated samples.

#### 6.1.2. *At laboratory accession:*

(4) Even if the existence of the centralized laboratory reception operated by a small expert staff is claimed to actually speed the handling of patient samples, the possibility of directly loading each section of the laboratory with its share of work, thus reducing possible causes of errors should be considered.

#### 6.1.3. *In the sample processing phase and result reporting:*

(5) It should be possible to load instruments with the original patient sample, without the need of splitting the patient sample into fractionated samples.

(6) In automated systems, the sample should be automatically identified at the time of sampling. There should be no need to follow a predefined loading sequence. Results should be automatically delivered to the information system. For instruments not provided with automatic identification of samples, external devices connected with the instruments (for instance bar-code light pen) should be used.

(7) For manual processing, work lists should be generated after automatic identification of samples.

#### 6.1.4. *In all the phases:*

(8) Manual transfer of identification, changes of identification code (for example a number instead of a name) and technology (for example from a bar-coded identification to a man-readable one) should be avoided, as well as the splitting of the patient sample into fractionated samples. In the laboratory, a clear record of identification transfers should be kept: their number, the identification of manual or automated transfer, the area of laboratory and the phase where they have been done, the change of identification code, and/or technology, if any, should be appropriately recorded. A similar record should be kept of manual identifications, if any.

### 6.2. *Adaptability*

Since the provision of health care occurs in a multiplicity of settings, the system should be capable of functioning in all of these.

### 6.3. *Compatibility*

While this document is focused upon the operation and role of clinical laboratory, this is only a part of the total process of health-care delivery. An optimal system for patient/sample or service linkage should be a harmonious component of the complete process of health-care delivery.

### 6.4. *Cost effectiveness*

It should be demonstrable that the system does not only provide the features previously listed, but also reduces the cost of its component function in the total process of health-care delivery.

### References

1. IUPAC International Union of Pure and Applied Chemistry, Analytical Chemistry Division and Clinical Chemistry Division. Nomenclature for automated and mechanised analysis. *Pure and Applied Chemistry*, **61** (1989).
2. Mieth, Von I. and Haeckel, R., *Journal of Clinical Chemistry and Clinical Biochemistry* **20** (1982).
3. Chambers, R. W., Rubin, M. I., *et al.* A positive donor-recipient identification system for a regional blood transfusion service. *Transfusion*, **13** (1973).

